# The crystal structure of mammalian inositol 1,3,4,5,6-pentakisphosphate 2-kinase reveals a new zinc-binding site and key features for protein function

**DOI:** 10.1074/jbc.M117.780395

**Published:** 2017-04-27

**Authors:** Elsa Franco-Echevarría, Julia Sanz-Aparicio, Charles A. Brearley, Juana M. González-Rubio, Beatriz González

**Affiliations:** From the ‡ Departamento de Cristalografía y Biología Estructural, Instituto de Química-Física “Rocasolano,” Consejo Superior de Investigaciones Científicas, Serrano 119, 28006 Madrid, Spain and; the § School of Biological Sciences, University of East Anglia, Norwich Research Park, Norwich NR4 7TJ, United Kingdom

**Keywords:** crystal structure, enzyme mutation, inositol phosphate, structure-function, zinc, IP5 2-kinase, inositol hexakisphosphate

## Abstract

Inositol 1,3,4,5,6-pentakisphosphate 2-kinases (IP_5_ 2-Ks) are part of a family of enzymes in charge of synthesizing inositol hexakisphosphate (IP_6_) in eukaryotic cells. This protein and its product IP_6_ present many roles in cells, participating in mRNA export, embryonic development, and apoptosis. We reported previously that the full-length IP_5_ 2-K from *Arabidopsis thaliana* is a zinc metallo-enzyme, including two separated lobes (the N- and C-lobes). We have also shown conformational changes in IP_5_ 2-K and have identified the residues involved in substrate recognition and catalysis. However, the specific features of mammalian IP_5_ 2-Ks remain unknown. To this end, we report here the first structure for a murine IP_5_ 2-K in complex with ATP/IP_5_ or IP_6_. Our structural findings indicated that the general folding in N- and C-lobes is conserved with *A. thaliana* IP_5_ 2-K. A helical scaffold in the C-lobe constitutes the inositol phosphate-binding site, which, along with the participation of the N-lobe, endows high specificity to this protein. However, we also noted large structural differences between the orthologues from these two eukaryotic kingdoms. These differences include a novel zinc-binding site and regions unique to the mammalian IP_5_ 2-K, as an unexpected basic patch on the protein surface. In conclusion, our findings have uncovered distinct features of a mammalian IP_5_ 2-K and set the stage for investigations into protein-protein or protein-RNA interactions important for IP_5_ 2-K function and activity.

## Introduction

Inositol 1,3,4,5,6-pentakisphosphate 2-kinase (IP_5_ 2-K)^3^ is a key enzyme of higher inositol phosphate (inositide, IP) metabolism. IP_5_ 2-K is present in yeast to mammals and catalyzes the synthesis of phytic acid (inositol hexakisphosphate or IP_6_) from IP_5_ and ATP (1). A variety of roles have been proposed for this enzyme and its product IP_6_ (2) in DNA repair (3), mRNA editing, export and degradation (4, 5), vesicle trafficking (6) and protein ubiquitylation (7). At a molecular level, IP_6_ acts as a cofactor for proteins with DNA-dependent protein kinase activity in non-homologous end joining (8) and with GLE1 in mRNA export (4). IP_6_ also can act as a folding factor as in the case of adenosine deaminase that participates in editing of mRNA and tRNA (5). In addition, IP_6_ is the precursor of inositol pyrophosphates, essential for cellular energy homeostasis, signal transduction control, and apoptosis (9, 10). Mice embryos with IP_5_ 2-K deletion do not survive more than a few weeks (11). More recently, a role in ribosomal rRNA synthesis independent of IP_5_ 2-K catalytic function has been proposed for the human enzyme (12), which has been shown to colocalize with mRNA either in the nucleus or cytoplasm (13). All these findings increase the potential of this enzyme as an attractive target.

IP_5_ 2-K belongs to the inositol polyphosphate (IPK) structural family that include enzymes capable of phosphorylating hydroxyls at different positions of the inositol ring starting from inositol 1,4,5-trisphosphate (IP_3_), a well known second messenger responsible for calcium mobilization (14). These phosphorylation events occur in combination with another family of inositol kinases that adopts an “ATP grasp-like” fold (15, 16). Both families together cover a great range of phosphorylation reactions on the six −OH positions of the *myo*-inositol ring and even on those already phosphorylated (15, 17, 18). Many of these enzymes present redundant abilities acting on similar substrates or they bind an inositide in different orientations that is thus phosphorylated in different positions (19). In contrast, IP_5_ 2-K is the unique IPK whose physiological role is the phosphorylation of the axial 2-OH position of *myo*-inositol, the other five hydroxyls being in equatorial positions. It is also a very specific enzyme and, together with IP_3_ 3-K, phosphorylates just one position of inositol (20, 21).

IPK enzymes are classified as a structural subgroup of the protein kinase (PK) family, because they conserve a few features including a fold in two separated lobes (N- and C-lobe) and similar nucleotide recognition mode (22). In addition, the core of the N-lobe and a few involved in catalysis are conserved. In 2004, the first structure of an IPK was described from IP_3_ 3-K (23). Since then, the structure of at least one member of each IPK subfamily has been reported (23–26). In summary, the IPK family presents a specific and characteristic fold in the C-lobe different from PKs, having a β-sheet core with helical insertions showing a great range of sizes depending on the IPK class. These helical regions are involved in substrate binding and have been named by us as IP-lobe or CIP-lobe (19). Thus, promiscuous IPKs, such as the IP multikinases, have a single helix inserted showing an open active site able to cope with various substrates and products, whereas more specific enzymes as IP_3_ 3-K and IP_5_ 2-K show larger helical scaffolds. In fact, IP_5_ 2-K shows the most elaborated helical region.

The full-length structure of IP_5_ 2-K from *Arabidopsis thaliana* (*At*IP_5_ 2-K) is the only one known for this subfamily (25). It shows the features described above and shows that it is a zinc metallo-enzyme, for which a structural role has been proposed (25). We subsequently captured different IP_5_ 2-K conformations by X-ray crystallography (27) displaying open, half-closed, or closed conformations, as the nucleotide and/or the inositide are absent or present in the active site. Extensive work by others and us has identified the residues involved in substrate recognition and catalysis and has depicted the participation of the N-lobe in achievement of a productive conformation (25, 27–30). The structure of *At*IP_5_ 2-K was an important advance for this field, but nevertheless, the specific features of mammal IP_5_ 2-K remain unknown. The IP_5_ 2-K family shows moderate sequence conservation across the species. In particular, mammalian enzymes show different insertions and do not conserve the zinc site found in the plant enzyme, making it difficult to obtain a good sequence alignment. From its sequence motifs, putative zinc-binding residues have been proposed in human IP_5_ 2-K (13). However, if mammal IP_5_ 2-K is a zinc metallo-enzyme, the putative role for this metal awaits further studies. Because of the significance of IP_5_ 2-K to proper cell functioning and the many roles of its product IP_6_, it is important to understand the molecular basis that underlies this enzyme function. In this work, we have determined the mouse IP_5_ 2-K structure in the presence of inositide showing that although this enzyme conserves features with the plant enzyme, it differs significantly in many aspects. Our results define the specific features of mammal IP_5_ 2-Ks. In addition, we present here valuable information that could help in understanding IP_5_ 2-K functions beyond its catalytic activity as its role in ribosomal RNA synthesis (12).

## Results

### Structure of IP_5_ 2-K mammalian isoform

We have solved the structure of *Mus musculus* IP_5_ 2-K (*m*IP_5_ 2-K) at 2.4 Å resolution (Table 1) from a truncated form lacking the 21 C-terminal residues (ΔC-*m*IP_5_ 2-K). Noticeably, *m*IP_5_ 2-K crystals were not obtained in the absence of the inositide. The structure for *m*IP_5_ 2-K in the presence of one or both ligands, forming binary complexes (IP_6_) or ternary complexes (IP_5_ + ATP), is presented (supplemental Fig. S1). In addition, we present two different crystal forms, including one or two molecules in the asymmetric unit, respectively, the first showing much better resolution (2.4 *versus* 3.2 Å). As mouse and human IP_5_ 2-K isoforms share 91% of sequence identity, we propose the structure of the mouse enzyme as a template for the mammalian IP_5_ 2-Ks.

**Table 1:** **Crystallographic data statistics and refinement**

	ΔC-mIP_5_ 2-K	ΔC-mIP_5_ 2-K	ΔC-mIP_5_ 2-K
Ligand modeled	+IP_5_ + ATP	+IP_6_	+IP_5_ + ATP
Crystallization pH	6.25	5.50	5.50

**Data collection and processing**			
Space group	P2_1_	P2_1_	P2_1_
Unit cell *^a, b, c^* (Å)	64.62, 140.76, 68.66	60.55, 71.64, 61.82	60.16, 71.50, 61.20
Unit cell α, β, γ (Å)	90.0, 106.5, 90.0	90.0, 111.7, 90.0	90.0, 111.4, 90.0
Temperature (K)	100	100	100
Radiation source	Synchrotron	Synchrotron	Synchrotron
Wavelength (Å)	0.979490	0.979260	0.979260
Resolution range (Å)	49.86–3.20 (3.42–3.20)	44.82–2.54 (2.65–2.54)	71.50–2.40 (2.49–2.40)
No. of observed reflections	134,008 (24,350)	106,382 (13,008)	123,314 (13,608)
No. of unique reflections	19,318 (3482)	16,270 (1970)	18,528 (1961)
Multiplicity	6.9 (7.0)	6.5 (6.6)	6.7 (6.9)
Data completeness (%)	99.3 (99.0)	99.6 (99.7)	97.3 (99.8)
Matthews coefficient (Å^3^ Da^−1^)	2.32	2.32	2.30
No. of molecules in a.u.	2	1	1
Wilson *B-*factor (Å ^2^)	83.26	54.65	45.72
Mean *I*/σ (*I*)	14.7 (3.3)	12.5 (3.1)	14.9 (3.5)
*R*_merge_ (%)*^a^*	10.4 (63.5)	7.6 (58.8)	7.3 (52.8)
*R*_pim_ (%)*^b^*	4.3 (25.8)	3.2 (24.6)	3.1 (21.5)
*CC*1/2	0.99 (0.89)	0.99 (0.99)	0.99 (0.97)

**Refinement**			
Resolution range (Å)	70.37–3.20	57.45–2.60	56.99–2.40
*R*_work_/*R*_free_*^c^* (%)	22.51/24.53	25.34/28.95	24.10/27.12
No. of atoms/*B*av (Å^2^)	6647/103.43	3348/69.36	3451/52.81
Protein	6575/103.15	3285/69.65	3342/53.178
Ligand	63/153.38	36/64.72	63/44.77
Zinc	4/79.47	1/46.91	1/49.87
Magnesium	2/102.48		2/38.56
Water molecules	3/51.26	26/39.60	39/35.34
Ramachandran plot (%)			
Favored/outliers	88.8/0.0	92.8/0.3	91.7/0.3
r.m.s.d.			
Bonds/angles (Å/°)	0.007/1.18	0.008/1.29	0.006/1.22
Protein Data Bank codes	5MWL	5MWM	5MW8
Missing residues in Protein Data Bank	A: 1–4/99–102/221–225/296–311/413–426/465–468	1–9/40–41/122–128/221–228/244–248/298–310/411–419/465–468	1–4/41–42/122–128/221–228/244–245/296–310/411–419/465–468
	B: 1–7/36–43/97–105/221–226/244–251/277–278/298–311/412–426/464–468		

*^a^ R*_merge_ = Σ*_hkl_* Σ*_i_*|*I_i_*(*hkl*) − (*I*(*hkl*))|/Σ*_hkl_* Σ*_i_ I_i_*(*hkl*), where *I_i_*(*hkl*) is the measurement of reflection *hkl* and (*I*(*hkl*)) is the weighted mean of all measurements.

*^b^ R*_pim_ = Σ*_hkl_* (1/(*N*-1)) 1/2 Σ*_i_*| *I_i_*(*hkl*) − (*I*(*hkl*))|/Σ*_hkl_* Σ*_i_ I_i_*(*hkl*), where *N* is the redundancy for the *hkl* reflection.

*^c^ R*_work_/*R*_free_ = Σ*_hkl_*|*F_o_* − *F_c_*|/Σ*_hkl_*|*F_o_*|, where *F_c_* is the calculated and *F_o_* is the observed structure factor amplitude of reflection *hkl* for the working/free (5%) set, respectively.

**Figure 1.**
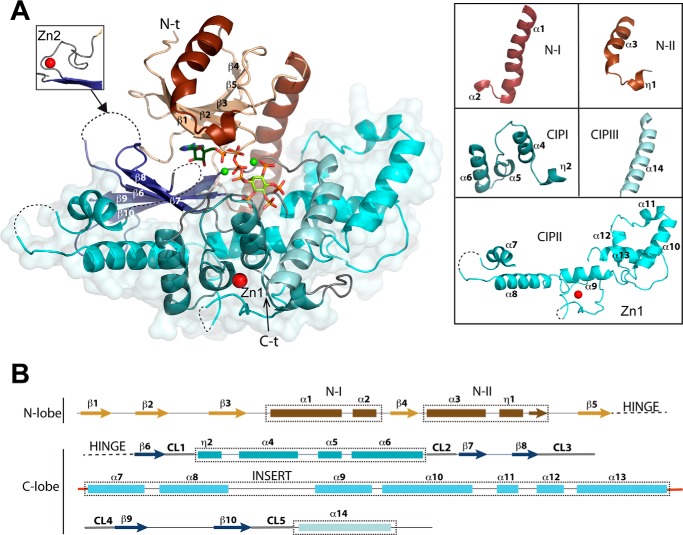
**Structure of *m*IP_5_ 2-K.** *A,* schematic representation of the structure of *m*IP_5_ 2-K. The N-lobe and C-lobe are shown in *orange-brown* and *blue* colors, respectively. ATP and IP_5_ are shown as *green sticks*, highlighting the oxygen, nitrogen, and phosphorus atoms in *red, blue,* and *orange*, respectively. The zinc and magnesium ions are shown as *red* and *green spheres*, respectively. The *left inset* shows a second zinc site found in one of the complexes. The *dashed lines* show disordered regions. On the r*ight,* the five helical segments found in *m*IP_5_ 2-K are detailed. *B, arrows* indicate β-strands and *rectangles* the α-helices. The connecting loops between C-lobe β-sheet and CIP-lobe are highlighted in *gray*. Conserved sequence motifs within the IPK family are concentrated in the CL β-strands connections (CL1, ^136^EIKPK; CL2, ^206^QNN*X*R*X*F; CL3, variable in sequence and length; CL4, ^400^DCSIMI; and CL5, ^436^LDLDLK).

**Figure 2.**
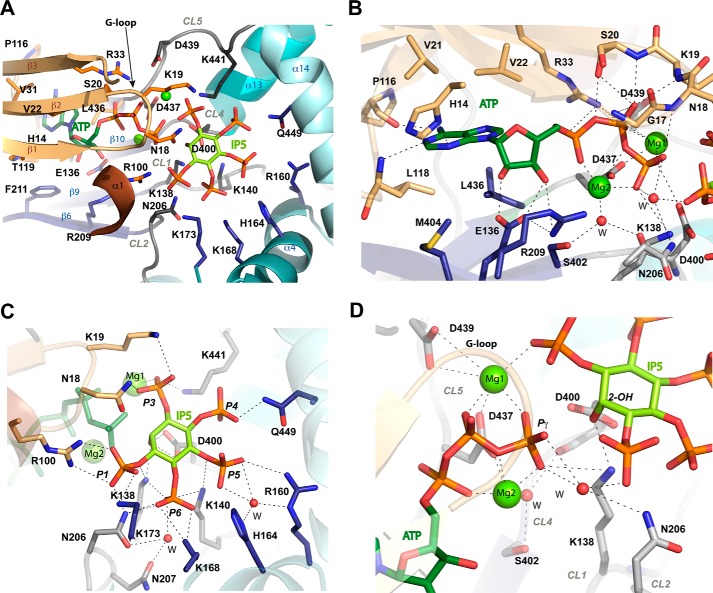
**Substrate recognition by *m*IP_5_ 2-K.** *A,* zoom of Fig. 1*A* showing the substrates ATP and IP_5_ and the residues involved in their recognition as sticks in *light orange* (N-lobe), *blue* (C-lobe), and *gray* (CLs in C-lobe). *B,* zoom showing the nucleotide site. Water molecules are shown as *red spheres. C,* zoom showing the inositide site. *D*, zoom showing inositide and nucleotide interaction.

Mouse IP_5_ 2-K folds in two lobes, N- and C-terminal lobes, connected by a hinge, thereby conserving the general fold scheme of PKs and IPKs, and in a similar way, both lobes coordinate the nucleotide between them (Fig. 1*A*). The N-lobe core forms a β-sheet formed by five antiparallel β-strands (β1–β5) showing two helical segments. The first helical segment (N-I) harbors α1, equivalent to the helix αC characterized in all protein kinases, whereas the second one (N-II) is a specific insertion different in every IPK subfamily. A role of this region for substrate binding in the IP_5_ 2-K subfamily has been previously reported by others (29) and by us (25, 27). Regarding the C-lobe, it also presents a β-sheet core formed by five antiparallel β-strands (β6–β10). Three helical segments are inserted in the β-sheet core. These segments altogether form a large helical ensemble named the CIP lobe in the structure of *At*IP_5_ 2-K (25), and each of them is consequently named as CIP_I_, CIP_II_, and CIP_III_. The CIP lobe represents more than half of the protein and is specific to IP_5_ 2-K enzymes. It creates a scaffold that builds up most of the inositide substrate-binding site. The five loops (CL1–CL5) joining the CIP region to the C-lobe β-sheet core are essential because they play a key role in substrate binding and catalysis (Figs. 1*B* and 2).

### mIP_5_ 2-K active site and substrate recognition

A general view of IP_5_ 2-K substrate recognition is shown in Fig. 2*A* and detailed in supplemental Table S1. The adenine is strongly recognized through polar and hydrophobic interactions with both protein lobes and the hinge connecting them (Fig. 2*B*). In particular, it forms polar interactions with His-14 and the backbones of Pro-116 and Leu-118. The ribose OHs interact with the C-lobe residues Glu-136 and Arg-209. The triphosphate moiety is tightly bound to the N-lobe of the enzyme through polar interactions and to the C-lobe through two magnesium ions. In particular, phosphate interaction with residue Arg-33, with a flexible loop (G-loop, residues Gly-15–Ser-20) and with an acidic residue (Asp-437) through the magnesium ions, is conserved throughout the PK superfamily and is essential for nucleotide binding and kinase activity.

**Figure 3.**
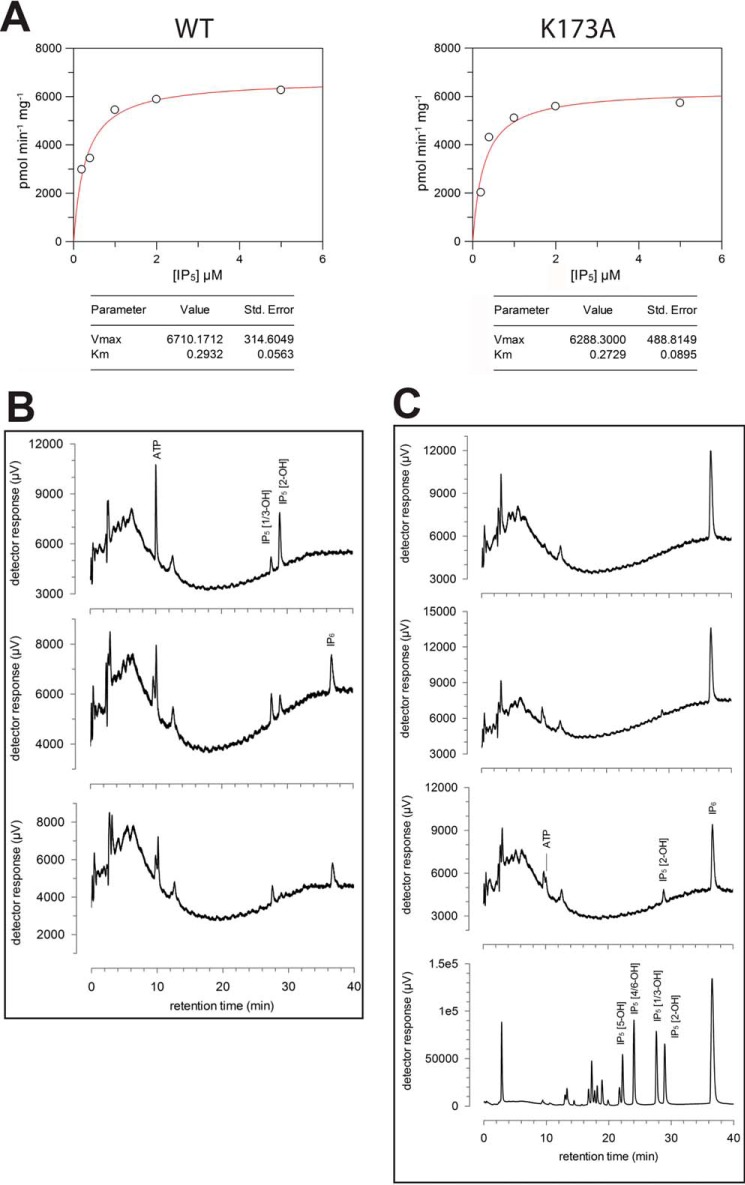
**Enzyme assays and reaction catalyzed by *m*IP_5_ 2-K samples.** *A*, kinetic parameters of LSLt-mIP_5_ 2-K WT and K173A variant. *B,* kinase reaction catalyzed by *m*IP_5_ 2-K. Products of enzyme assays resolved by ion-exchange HPLC: substrate, Ins(1,3,4,5,6)P_5_ (IP_5_) (*upper panel*); and reaction products after 60 min (*middle panel*) and 260 min (*lower panel*) of incubation of IP_5_ and ATP with enzyme. *C*, reversibility of reaction catalyzed by *m*IP_5_ 2-K. Products of enzyme assays resolved by ion-exchange HPLC: substrate, IP_6_ (*upper panel*), reaction products after 110 min (*upper middle panel*) and 310 min (*lower middle panel*) of incubation of IP_6_ and ADP with enzyme. The *lower panel* shows IP standards obtained by acid hydrolysis of IP_6_.

Regarding the inositide substrate, IP_5_ is tightly bound to the enzyme through its five phosphates (Fig. 2*C*). A total of 14 residues coordinate the phosphate groups, two of them through water molecules and eight of them being lysine or arginine. P1 and P3 are coordinated by both lobes, whereas P4 and P5 are coordinated exclusively by the C-lobe. Residues from the N-lobe involved in P1 and P3 binding come from the segment N-II (Arg-100) and the G-loop (Asn-18 and Lys-19) mentioned above. Residues from the C-lobe involved in coordination of the five phosphates come from the CIP lobe and its CLs. Fig. 2*C* and supplemental Table S1 show all the polar interactions produced with the inositide phosphates (P1: Asn-18, Arg-100, Lys-138, Lys-173, and Asn-206; P3: Asn-18, Lys-19, and Lys-441; P4: Gln-449; P5: with Lys-140, Arg-160, and His-164 through water molecules; and P6: Lys-138, Lys-140, Lys-168, Asn-206, and water mediated with Asn-207). The tight and extensive recognition explains the high specificity of this enzyme. Human IP_5_ 2-K displays a 0.43 μm *K_m_* value for IP_5_ (31) in good agreement with the value obtained for *m*IP_5_ 2-K by us (0.29 μm) (Fig. 3*A*) and in contrast with the low tens of micromolar values reported for the *At*IP_5_ 2-K (22 μm) (32). A possible explanation for the different *K_m_* values found in the plant enzyme might be related to some differences found in enzymes from both kingdoms either in the inositide recognition mode or in the constraints introduced by the zinc-binding site (see below).

Six residues from the CLs form the interface of substrate recognition, generating a net of interactions, including the magnesium ions (Fig. 2*D*). Residues Lys-138 and Asp-400 make direct interaction with Pγ and/or the nucleophile 2-OH. The distance between the 2-OH and Pγ oxygen is 3.2 Å suggesting an in-line transference mechanism probably in agreement with an associative mechanism. Pγ is oriented through a magnesium ion (Mg1) coordinated to Asp-437 and to Asp-439 in a second sphere. Asp-437 also coordinates a second magnesium ion (Mg2) together with Ser-402 through a water molecule. Comparison of the ternary and binary complexes (IP_5_ 2-K/IP_5_/ATP *versus* IP_5_ 2-K/IP_6_) shows that there is no significant structural variation among them (r.m.s.d. is 0.377 Å for 403 Cα atoms). The IP_6_ shows similar interactions, with P2 remaining at the substrate interface regions described above. A BLAST search using *m*IP_5_ 2-K sequence and limited to mammals (taxid: 40674) shows that all residues coordinating the nucleotide and inositide are absolutely conserved, with just a couple of residues showing a conservative change in some species.

Unexpectedly, the formation of the ternary complex (*m*IP_5_ 2-K + IP_5_ + ATP) was achieved by protein incubation with IP_6_ and ADP (supplemental Fig. S1). Therefore, we checked that our crystallized *m*IP_5_ 2-K samples are able to catalyze both the forward (Fig. 3*B*) and reverse (Fig. 3*C*) reactions in solution. In agreement, the plant enzyme is highly reversible with an equilibrium constant in the forward “kinase” direction of ∼14 (20). By ion-pair reverse-phase HPLC, we were also able to confirm the production of ATP from IP_6_ and ADP (data not shown).

### Mammal IP_5_ 2-Ks share a zinc-binding site with a novel structure

**Figure 4.**
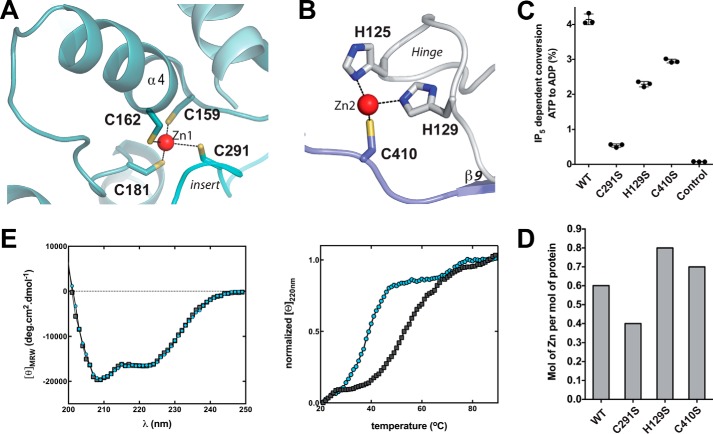
**Zinc-binding sites located in *m*IP_5_ 2-K complexes.** *A,* schematic representation of *m*IP_5_ 2-K Zn1 site, and *B*, of Zn2 site. *C*, plot of IP_5_-dependent conversion from ATP to ADP of *m*IP_5_ 2-K WT and mutants of the zinc-binding residues. *Error bars* show the standard deviation. *D,* zinc content of mutated *m*IP_5_ 2-K samples relative to WT *m*IP_5_ 2-K. *E*, far-UV CD spectra (*left*) and thermal denaturation followed by CD (*right*) of WT (*black squares*) and C291S (*blue circles*) ΔC-*m*IP_5_ 2-K.

*m*IP_5_ 2-K presents two zinc ions in its structure, one in the CIP-lobe (Zn1) and the other close to the hinge region (Zn2) (Figs. 1 and 4). In fact, Zn1 is present in all the crystals obtained, whereas Zn2 was only detected in the low resolution ΔC-*m*IP_5_ 2-K crystals in which the two molecules in the asymmetric unit interact through the hinge region probably fixing a conformation captured by crystallography (supplemental Fig. S2).

**Figure 5.**
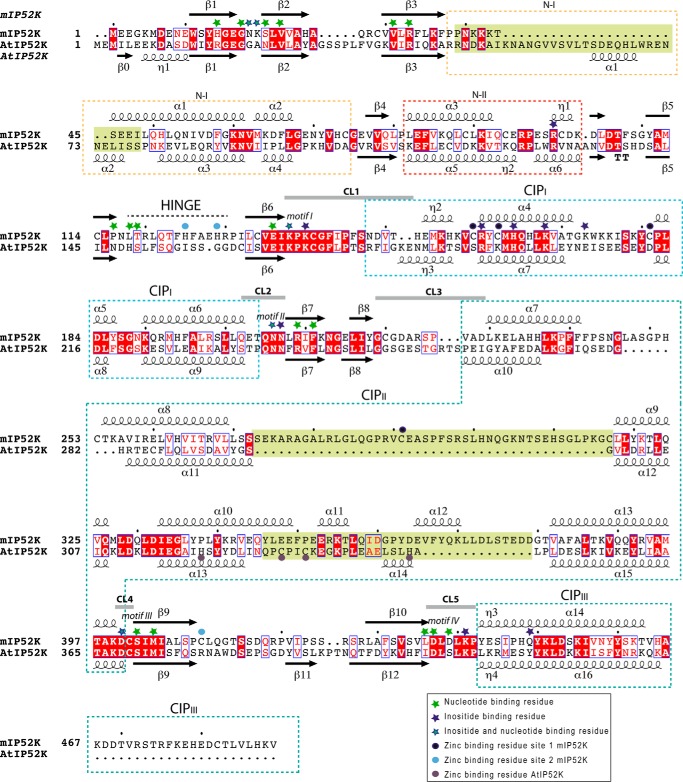
**Structural alignment of *m*IP_5_ 2-K *versus At*IP_5_ 2-K.** The secondary structure is shown at top (mammalian enzyme) and bottom (plant enzyme). Identical regions are *shaded red*, and similar regions are shown by *red letters. Light-green shaded squares* show regions with a divergent structure in both enzymes. Helical regions inserted into the β-sheet cores are marked with *dashed lined boxes*.

The Zn1 site is formed by residues from two CIP lobe elements: Cys-159, Cys-162, and Cys-181 from CIP-I and Cys-291 from CIP-II (Fig. 4*A*). This site presents the typical zinc geometry and coordination, although it has no homologues in the structural databases using the DALI server (33). The two first cysteines from CIP-I are located in a helix, separated by two residues, and could resemble a partial zinc-finger; however, the fourth ligand breaks any resemblance because it comes from a position very distant in sequence. In fact, Cys-291 comes from a very long loop inserted into two helices that cross over the back of the CIP-lobe (Fig. 5). We have selected Cys-181 and Cys-291 as candidates for the mutagenic study (Fig. 4*C*). However, there was no expression of the C181S *m*IP_5_ 2-K mutant in the conditions reported herein for the wild-type protein. This suggests that the Zn1 site formation could be essential for proper protein folding. By contrast, C291S *m*IP_5_ 2-K mutant expression levels are in the same order as the wild type, being only 2-fold decreased. Despite this, the impact of this mutation on enzyme activity is very high, because the mutated sample retains less than 10% of enzyme activity (Fig. 4*C*). In this line, it is worth mentioning that the two first cysteines of this zinc site are in helix α4, an element that provides four residues for the inositide substrate binding (Fig. 2*A*). We also consider that this fact could have some effect in obtaining a lower *K_m_* value for the substrate in *m*IP_5_ 2-K.

Regarding Zn2, it is created by two residues from the hinge (His-125 and His-129) and one residue from the C-lobe (Cys-410) (Fig. 4*B*). Mutation of residues His-129 and Cys-410 to serine produces samples with a moderate decrease in enzymatic activity revealing that Zn2 is not critical for protein function (Fig. 4*C*). A possible explanation for the greater decrease found in the H129S mutant could be its key location in the hinge, an element important for enzyme flexibility.

We have subsequently evaluated the metal content in *m*IP_5_ 2-K samples by inductively coupled plasma-optical emission spectroscopy (ICP-OES) (Fig. 4*D*). We confirmed that *m*IP_5_ 2-K is a zinc metalloenzyme (supplemental Table S2). Unexpectedly, wild-type samples purified as LSLt-tagged protein exhibit a zinc/protein molar ratio of 0.6:1. An insufficient supply of zinc could also explain the difficulties encountered in the crystallization process of this enzyme due to structural inhomogeneity. However, attempts to add zinc from different salts to the crystallization drops failed because the protein precipitated. We found a zinc/protein molar ratio of 0.8:1, 0.4:1, and 0.7:1 for H129S, C291S, and C410S, mutants, respectively. As expected, mutation on residue Cys-291 (Zn1 site) yields an enzyme with less zinc content than the wild type (Fig. 4*D* and supplemental Table S2). By contrast, mutation of Zn2-coordinating residues (His-129 and Cys-410) yields samples with no reduction in the zinc content (Fig. 4*D* and supplemental Table S2). Therefore, we further analyzed the features of the C291S mutant by circular dichroism (CD) (Fig. 4*E*). We found that both WT (wild type) and C291S *m*IP_5_ 2-K samples display a similar far-UV CD spectra suggesting that they share similar secondary structural elements. However, thermal denaturation followed by CD revealed that the mutated sample exerts an apparent *T_m_* (32 °C) drastically reduced compared with that of the WT sample (43 °C).

In conclusion, our results suggest that Zn1 is necessary for protein folding and stability. Furthermore, the reduced capacity of the C291S mutant to bind zinc correlates with a high reduction in protein activity (Fig. 4, *C* and *D*). In agreement, IP_5_ 2-Ks from mammals show conservation only in the Zn1 site, with its four cysteine ligands being fully conserved. Therefore, Zn1 is key in all mammal IP_5_ 2-K enzymes, whereas Zn2 could be an artifact of crystallization.

### Mammal and plant IP_5_ 2-Ks show three large structural divergences

Until now, the other IP_5_ 2-Ks with known structure is that from *A. thaliana* (25). A Cα superposition of *m*IP_5_ 2-K onto *At*IP_5_ 2-K (Protein Data Bank code 2xan) overlays 327 residues (out of 468 in *m*IP_5_ 2-K) with an r.m.s.d. of 1.2 Å. A good sequence alignment between both enzymes has remained elusive because their sequence homology is not very high (24% identity and 38% similarity), and they present different insertions. A structural alignment of both IP_5_ 2-K isoforms is shown in Fig. 5. Although the topology of the N- and C-lobe cores and substrate-binding region is quite conserved, both enzymes present multiple dissimilarities.

**Figure 6.**
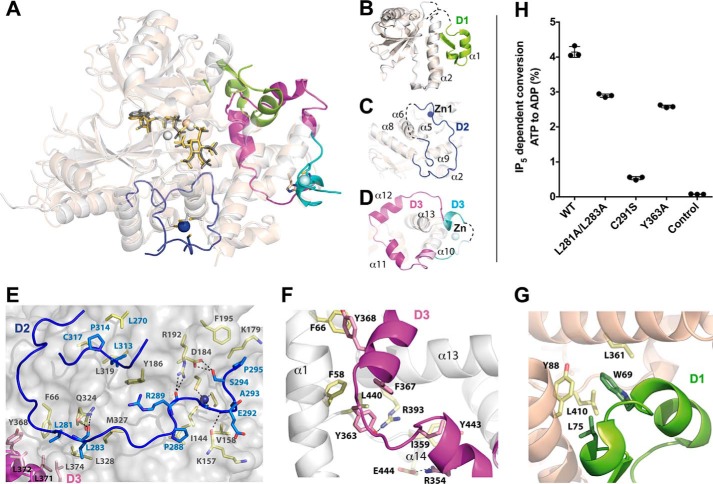
**Novel regions of *m*IP_5_ 2-K and structural comparison with *At*IP_5_ 2-K.** *A,* schematic representation of *m*IP_5_ 2-K (*white*) and *At*IP_5_ 2-K (*wheat*) superposed structures. Zinc ions are shown as *dark blue* (*m*IP_5_ 2-K) and *cyan* (*At*IP_5_ 2-K) *spheres*. Magnesium ions are shown as *spheres* in similar color to the isoform to which they belong. The main differences between both samples (D1, D2, and D3) are *highlighted* in different colors. *B,* representation of N-lobes from both isoforms highlighting D1 (*green*) insertion in *At*IP_5_ 2-K. *C,* piece of CIPII lobe showing D2 in *m*IP_5_ 2-K (*blue*). *D*, view showing D3 (*magenta* in *m*IP_5_ 2-K and *cyan* in *At*IP_5_ 2-K). *E,* intramolecular interactions produced by D2 in *m*IP_5_ 2-K. *Blue, pink,* and *yellow sticks* show residues from D2, D3, and the rest of the protein, respectively. *F,* intramolecular interactions produced by D3 in *m*IP_5_ 2-K. *G,* intramolecular interactions produced by D1 in *At*IP_5_ 2-K. *Green sticks* show residues from D1. *H,* plot of IP_5_-dependent conversion of ATP to ADP of WT *m*IP_5_ 2-K and several mutants of residues located in the different regions. *Error bars* show the standard deviation.

The most predominant divergences are found within three regions, which show large differences in their sequence, length, and topology (Figs. 5 and 6, *A–D*). The first main difference (D1) is located in N-I region, *At*IP_5_ 2-K having an insertion not present in the mammal enzyme (Fig. 6*B*). A possible function of this segment will be discussed below. The second clear difference (D2) is concentrated within the CIP-II region (Fig. 6*C*). Mouse IP_5_ 2-K presents a long and flexible loop (Ser-272–Gly-316, 45 residues) that crosses back from the whole CIP (Figs. 5 and 6*C*). Interestingly, this loop is the one that provides a cysteine residue (Cys-291) that completes the Zn1 site present in *m*IP_5_ 2-K (Fig. 4). In addition, this loop seems to stabilize the protein, because it packs with several regions of the enzyme, including both lobes, but mainly the CIP lobe. In particular, it makes hydrogen bonds through four residues (Leu-283, Arg-289, Glu-292, and Ser-294) and shows strong hydrophobic interactions through seven residues (Leu-281, Leu-283, Pro-288, Ala-293, Pro-295, Leu-313, and Pro-314) (Fig. 6*E*). These residues are fully or highly conserved along the mammal isoforms. Only residues Leu-283 and Arg-289 show a great variation, and both are involved in hydrogen bonds through their main chain atoms with other parts of the protein (Fig. 6*E*). Finally, a third difference (D3) is shown in a region also located in the CIP-II (Fig. 6*D*). Precisely, *At*IP_5_ 2-K presents a zinc site located in this region (25) that is shorter than and completely different from that found in *m*IP_5_ 2-K (Fig. 6, *C* and *D*).

Noticeably, different regions of the two isoforms seem to share roles. On the one hand, D1 and D3, from *At*IP_5_ 2-K and *m*IP_5_ 2-K, respectively (Fig. 6, *B* and *D*), are both stabilizing essential zones of the enzyme, as are α1 (Asn-54–Phe-66, analogous to protein kinases αC) and other CIP residues. Some relevant interactions of these regions are shown in Fig. 6, *F* and *G*. Among them, it is worth mentioning the central role of Tyr-363, Phe-367, and Tyr-368 in *m*IP_5_ 2-K (Fig. 6*F*), residues absolutely conserved in the mammal IP5 2-K isoforms. *At*IP_5_ 2-K presents residues with roles similar to Tyr-363 (Leu-75) and Phe-367 (Trp-69), which interact with equivalent regions of the protein (Fig. 6*G*). We have selected residue Tyr-363 for mutation, because it seems key in the network of interactions as proposed above. Y363A mutation halves enzymatic activity (Fig. 6*H*). Unexpectedly, this decrease in activity is moderate. An explanation is that it corresponds to a single mutation among a multiple net of interactions and in a residue far from the active site. On the other hand, *m*IP_5_ 2-K D2 and *At*IP_5_ 2-K D3 (Fig. 6, *C* and *D*) are both involved in generation of the zinc sites, which are not conserved either in sequence, location, or structure between both enzymes. However, both sites seem to have a structural role, although we cannot discard any other additional function. Strikingly, the insertions found in *m*IP_5_ 2-K (D2 and D3) interact with each other (Fig. 6*E*). Thus, Tyr-368 in *m*IP_5_ 2-K and the following leucine residues (Leu-372 and Leu-374) interact with Leu-281 and Leu-283 located in D2 (Fig. 6*E*). A double mutation in this region (L281A/L283A) reduces slightly the activity in contrast with the >90% decreases caused by the other mentioned mutation on a zinc-binding residue (C291S) within this segment (Fig. 6*H*). All the mentioned leucine residues show very high conservation, except Leu-283, as mentioned previously. A mutation in this residue would keep the capacity of making hydrogen bonds through its main chain (Fig. 6*E*) and the slight structural destabilization introduced has no impact in the protein-active site.

### Novel findings in mammal IP_5_ 2-K substrates binding and catalysis

**Figure 7.**
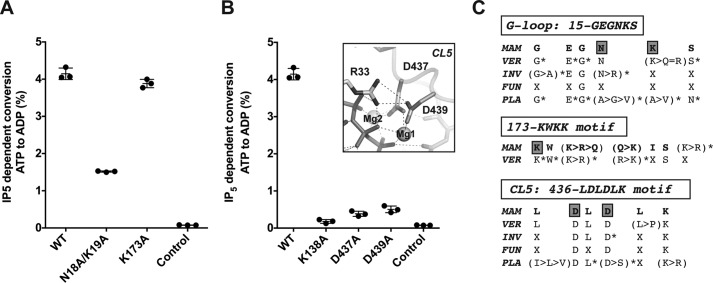
**Analysis of relevant residues for *m*IP_5_ 2-K function.** *A,* plot of IP_5_-dependent conversion of ATP to ADP by *m*IP_5_ 2-K WT and several mutants to determine the effect of selected inositide-binding residues. *Error bars* show the standard deviation. *B,* same as *A* to check the effect of catalytic residues. The *inset* illustrates the salt bridge between Asp-439 and Arg-33. *C,* IP_5_ 2-K sequence conservation across the kingdoms in regions containing the mutated residues (Asn-18 and Lys-19; Arg-173 and Asp-439). Letter *X* and *asterisks* denote residues non-conserved or mostly full conserved, respectively. Abbreviations: *MAM,* mammals; *VER,* vertebrates other than mammals; *INV,* invertebrates; *FUN,* fungi; *PLA,* plants.

Most residues involved in substrate binding and catalysis are conserved between mammal and plant IP_5_ 2-Ks (Fig. 5). Nevertheless, we can observe some differences in the inositide P1 and P3 coordination. As shown, inositide P1 interacts extensively with Arg-100 of *m*IP_5_ 2-K (Fig. 2*C*). The role of the Arg-100 equivalent in *At*IP_5_ 2-K (Arg-130) has been largely argued, and an implication in substrate binding and triggering of a productive protein conformation has been proposed (27, 29, 30). Unfortunately, a construct prepared for the R100A *m*IP_5_ 2-K mutant did not show expression in the soluble fraction. However, we can conclude that this residue is structurally similar to Arg-130 in *At*IP_5_ 2-K. In *m*IP_5_ 2-K, additional interactions with P1 are produced through the side chain of Lys-173, a residue non-conserved with the plant IP_5_ 2-Ks but absolutely conserved in mammal enzymes, whereas conservative substitutions can be observed in other vertebrates (Fig. 7). However, mutation of Lys-173 produces an enzyme as active as the wild type (Fig. 7*A*) showing very similar kinetic parameters (Fig. 3*A*), suggesting that this interaction is dispensable for substrate binding and it probably might have other implications, as will be commented on later.

Inositide P3 coordinates with the main chain of the G-loop in both enzymes, but different additional interactions are made within the two enzymes. In *m*IP_5_ 2-K, P3 also interacts with the side chains of Asn-18 and Lys-19 G-loop residues, whereas the plant enzyme provides two arginine residues from a different region (Arg-45 and Arg-415) to complete this binding. The double mutation on the G-loop residues Asn-18/Lys-19 (Fig. 7*A*) has a notable impact in the enzymatic activity, supporting a main role for these G-loop residues absolutely conserved in mammals (Fig. 7*C*).

We also can observe particular features in catalytic residues in the connecting loops (Fig. 1*B*). We have prepared mutations on relevant residues from these CLs (K138A, D437A, and D439A) (Fig. 2*D*), obtaining mutated enzymes with very low activities (Fig. 7*B*). The role of equivalent residues to Lys-138 and Asp-437 has been largely studied along the PK and IPK families. These residues are responsible for neutralizing the negative charge developed in the transition state and orienting the nucleotide Pγ through magnesium ions, respectively. We observe in *m*IP_5_ 2-K that Asp-439 displays a strategic position, helping with magnesium coordination and forming a bridge with Arg-33, a residue involved in the coordination of nucleotide phosphates. Therefore, it seems to provide a proper conformation for essential parts of the enzyme (Fig. 7*B*). No mutagenesis data have been reported on the Asp-439 equivalent residues in other enzymes, because a homologous residue in this position is not present (neither in the IPK or PK families). Asp-439 shows a notable conservation in the whole IP_5_ 2-K family across the species (Fig. 7*C*), showing only changes by a serine residue in some plant IP_5_ 2-Ks, which in turn also coordinates the magnesium atom (25). We show here that the D439A mutation produces nearly inactive enzyme. Finally, the connecting loop CL3 does not show significant conservation between *m*IP_5_ 2-K and *At*IP_5_ 2-K, either in length or sequence. This loop is responsible in *At*IP_5_ 2-K for interactions between the N- and C-lobe that might partially regulate the catalysis through opening and closing the active site (27). In addition, this loop makes interactions with two residues directly involved in inositide binding, one of them (Arg-130) shown to be essential for protein activity (27). We think that differences in this loop may also account for the differences observed in the *K_m_* value from each protein. In the *m*IP_5_ 2-K structure, this loop is disordered, and therefore interaction with the N-lobe has not been determined (Fig. 1). However, the flexibility found in the *m*IP_5_ 2-K CL3 loop is consistent with a dynamic role and the previous proposed functions in catalysis regulation.

### Prominent basic patch on mIP_5_ 2-K surface

**Figure 8.**
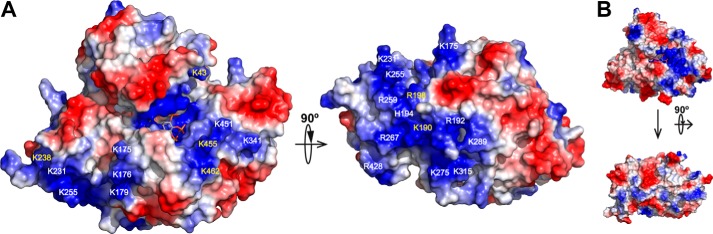
**Basic patch on *m*IP_5_ 2-K surface.** *A,* electrostatic surface representation of *m*IP_5_ 2-K showing its exposed basic patch. Residues conserved and non-conserved with the *At*IP_5_ 2-K are shown in *yellow* and *white,* respectively. *B,* same representation as in *A* for the *At*IP_5_ 2-K enzyme.

The structure of *m*IP_5_ 2-K shows a very notable basic patch on its surface, mainly concentrated down the active-site face of the enzyme (Fig. 8*A*). The enzyme regions that contribute to this patch are α6 and α8 in CIP-I and the large insert found in the CIP-II region. Interestingly, only a few of these basic residues are present or conserved in *At*IP_5_ 2-K (Fig. 8*B*), which apart from the basic pocket for the inositide binding does not show any significant accumulation of arginine or lysine residues on its surface. By contrast, most of these residues are conserved across the mammalian isoforms. This striking feature could be correlated with mammalian IP_5_ 2-K localization and/or other possible functions that this enzyme could present in cells. Particularly outstanding are two basic segments, one formed by residues Lys-175, Lys-176, and Lys-179, and the other containing the residues in the new insert (D2) found in mammals and its preceding helix (Lys-255, Arg-259, Arg-267, Lys-275, Lys-289, and Lys-315). The first segment overlaps in part with the motif ^173^KW(K/R)(K/Q), which is highly conserved in mammals. The second segment encloses one of the main differences (D2) found with respect to plant enzymes. Based on the present finding, we propose that this insert, apart from supporting the zinc binding and stabilizing some protein regions, could be delineating a particular protein surface that could serve as an interface for other partners essential for IP_5_ 2-K function.

## Discussion

Here, we described the first structure of a mammalian IP_5_ 2-K, a key enzyme in inositol metabolism with multiple impacts in diverse cellular events. Structural knowledge of this enzyme is essential to fully understand its function, although the fact that mammalian IP_5_ 2-K shows extremely low bacterial expression and a very low tendency to produce suitable crystals has precluded it until now. The structure of *m*IP_5_ 2-K presented here shows large structural differences with the *At*IP_5_ 2-K concentrated in the helical regions. Both enzymes are zinc metalloenzymes, the zinc sites showing a different location and structural features. Whereas the plant zinc site is exclusive for its kingdom, the site found in mammals (Zn1) seems to be conserved in all the species except plants. Point mutations of the Zn1 ligands present different effects, going from a null protein expression (Cys-181) to a decreased zinc amount (Cys-291) clearly correlated with a dramatic drop in protein activity and thermal stability. In particular, Cys-291, located in a long insertion (D2), is present in all vertebrates and most invertebrates (data not shown). This insertion is quite unusual because it is unstructured and placed between two contiguous helices (Fig. 6*C*). Its role seems to support the architecture of the CIP lobe to complete the zinc-binding site and to configure a markedly basic protein surface.

Apart from these remarkable differences found, substrate recognition in both isoforms is quite similar. In previous works, *At*IP_5_ 2-K was shown to display conformational changes upon substrate binding that set the enzyme lobes together producing a closed conformation (27). Studies performed with *At*IP_5_ 2-K revealed that the IP_5_ binding to the C-lobe is stronger, whereas the binding to the N-lobe is necessary for protein activation and conformational change (30). As the Zn1 is located in the CIP-lobe and far from the N-lobe, we suggest that it would affect the preliminary inositide binding events rather than the subsequent dynamic behavior of the protein. The structure solved for *m*IP_5_ 2-K probably corresponds to the close conformation in agreement with the fact that the inositide substrate is present in the structure and the good superposition of *m*IP_5_ 2-K reported here onto the *At*IP_5_ 2-K closed conformation. We do not know whether the changes reported for *At*IP_5_ 2-K also occur in the mammal isoforms and whether they are a general behavior of the IP_5_ 2-K family. However, the conservation of several elements involved in these changes, as the flexibility of CL3 or inositide binding by N-lobe through Arg-100 or G-loop, suggests that this open-close mechanism could be proposed for mammal IP_5_ 2-Ks. In relation to this, we made proteolysis experiments to check whether the substrates protect the enzyme digestion as happened in *At*IP_5_ 2-K (28). We observed no protection in *m*IP_5_ 2-K, probably due to the fact that its digestion sites are far from regions involved in the conformational change. Moreover, we did not get crystals in the absence of substrates, which could be pointing to some structural changes, although this is not conclusive either.

The structure of *At*IP_5_ 2-K revealed that IP_5_ 2-Ks are the most divergent among the IPK family, because it has the most elaborated CIP-lobe and binds the substrate in a very different orientation to face an axial OH to Pγ of phosphate. Similarly, IP_5_ 2-Ks are the most divergent enzymes classified inside the PK structural superfamily, which is confirmed in the presented structure. Moreover, we observe that *m*IP_5_ 2-K lacks the N-lobe acidic residue (Glu-91, PKA nomenclature) reported to salt-bridge to a basic residue (Lys-72, PKA nomenclature) and involved in ATP phosphate coordination. This bridge is a hallmark that identifies the active conformation of protein kinases, and equivalent residues are also present in other IPKs. In contrast, *m*IP_5_ 2-K has a different acidic residue, Asp-439, which stabilizes Arg-33 (equivalent to Lys-72 in PKAs). Therefore, in the IP_5_ 2-K family, this bridge could play a similar role to that described in PKs.

Brehm *et al.* (12, 13) have investigated the human (*h*) IP_5_ 2-K and, in particular, the possibility of additional functions apart from its catalytic role. They found that *h*IP_5_ 2-K colocalizes with mRNA, both in the nuclei and cytoplasm (13). The mutations on a region rich in basic residues, coincident with the ^173^KW(K/R)(K/Q) motif conserved in mammals, show an altered enzyme ability to be exported out of the nuclei. This region is exposed and included within the basic patch of *m*IP_5_ 2-K identified in this work (Figs. 7*C* and 8). Interestingly, Lys-173 at the beginning of the above motif coordinates the inositide substrate. However, our mutagenesis experiments showed that this residue is dispensable for substrate binding, suggesting that the major role for this motif is played in the translocation process proposed by Brehm *et al.* (13). In addition, *h*IP_5_ 2-K was shown to be a structural component of the nucleolus acting as a molecular scaffold in nucleoli and influencing the degree of rRNA synthesis, thereby having a role in rRNA biogenesis. In relation to this, *h*IP_5_ 2-K interacts with three proteins (CK2, TCOF, and UBF) that regulate rRNA synthesis. In particular, UBF interacts with a basic region (^41^RKK motif equivalent to ^41^KKK motif in *m*IP_5_ 2-K) (12) which mutation prevents the UBF translocation out of nucleolus after *h*IP_5_ 2-K overexpression. As the authors predict, this region is completely exposed, and we observed that it is in a flexible loop as shown by its poor electron density. We now disclose the high and specific basic region found in the mammalian IP_5_ 2-K surface that could be used as a guide to find more target points.

In conclusion, our work provides novel features for the IP_5_ 2-K family and its mammalian isoforms. Unexpected protein regions and residues have been identified providing an illuminating picture of these enzymes. The findings comprise the characterization of subtle but important features for substrate recognition, including unreported catalytic residues for this family, the identification of an unusual and exclusive zinc-binding site, and the conspicuous basic patch on the protein surface. Undoubtedly, the results obtained in this work provide a valuable tool for the design of therapeutics targeted at mammalian IP_5_ 2-K with potential implications in health and also to perform IP_5_ 2-K functional studies. Beyond the catalytic function, our work also suggests putative regions of interaction of mammalian IP_5_ 2-Ks with the cognate partners necessary to accomplish their precise functions.

## Experimental procedures

### Protein expression and purification

Constructs for full-length IP_5_ 2-K recombinant expression either in bacteria (m*ipk1*/pKLSLt plasmid) or insect cells were obtained, as described by us (34), from a m*ipk1* cDNA (commercial clone bc062167). To produce a truncated *m*IP_5_ 2-K enzyme lacking the 21 C-terminal residues (ΔC-*m*IP_5_ 2-K), a stop codon was introduced at a position coding for residue 469 of *m*IP_5_ 2-K by site-directed mutagenesis and using as template the m*ipk1* cDNA inserted into the pKLSLt vector (35). Point and double ΔC-*m*IP_5_ 2-K mutants were obtained by site-directed mutagenesis using as template the ΔC-m*ipk1*/pKLSLt plasmid. Primers used for construct preparations are shown in supplemental Table S3.

Expression and purification of ΔC-*m*IP_5_ 2-K samples fused to LSL− was performed similarly to the full-length samples (34). Briefly, the protein was expressed in *Escherichia coli* BL21 Star (DE3) cells in 2TY medium supplemented with kanamycin (50 μg ml^−1^) at 310 K until an *A*_600_ of 0.9 was reached. Expression was induced with 0.3 mm isopropyl 1-thio-β-d-galactopyranoside for 96 h at 283 K. Pellets were resuspended and sonicated in buffer A (20 mm Tris-HCl, pH 8.0, 150 mm NaCl, 1 mm DTT) plus 0.2 mm PMSF and 0.05% Triton X-100. The filtrated lysate was diluted 3-fold, loaded onto a heparin column, washed with buffer B (20 mm Tris-HCl, pH 8, 50 mm NaCl, 1 mm DTT), and eluted with a 1 m NaCl gradient. The fusion protein was applied to a Sepharose CL-6B column equilibrated in buffer A and eluted using 200 mm lactose, followed by overnight cleavage with TEV protease (protease/protein mass ratio 1:40) gently rolling at 278 K. Our protein was separated from LSLt and TEV protease by a second heparin column and further purified by size-exclusion chromatography (HiLoad 16/600 Superdex 200 column) equilibrated in buffer A plus 2 mm IP_6_, which was included to avoid protein precipitation. All *m*IP_5_ 2-K samples used for crystallization were concentrated to around 5–6 mg ml^−1^ and stored at 193 K. We obtained 1 mg of pure ΔC*-m*IP_5_ 2-K per liter of bacteria culture. The purity of all the samples was confirmed by SDS-PAGE. For crystallization and CD analysis, WT and C291S ΔC-IP_5_ 2-K samples were purified using this protocol.

Finally, for activity assays, wild-type LSLt-ΔC-*m*IP_5_ 2-K and mutants were purified as follows. Clarified and filtrated cell lysate in buffer A was applied to a Sepharose CL-6B column equilibrated in buffer A. After washing with buffer A, the protein was eluted with 200 mm lactose. The sample was diluted 3-fold with 20 mm Tris-HCl, pH 8.0, loaded onto a heparin column, and washed with buffer B and eluted with a salt gradient. The protein in final buffer C (20 mm Tris-HCl, pH 8.0, 700 mm NaCl, 1 mm DTT) was concentrated to 1–3 mg ml^−1^ and stored at −80 °C.

### Crystallization

All IP_5_ 2-K samples used for crystallization were obtained in the presence of 2 mm IP_6_. Best crystals obtained for the full-length *m*IP_5_ 2-K (expressed either in bacteria or in insect cells) diffracted to 4–4.3 Å (34), and they did not allow the structure solution. Finally, the ΔC-*m*IP_5_ 2-K construct allowed us to improve resolution to 3.2 Å from crystals grown in 0.2 m magnesium chloride, 0.1 m MES, pH 6.25, 10% (v/v) PEG 6000 and included 2 mm IP_6_ and 2 mm ADP in the protein buffer. A new pH grid screen using the sample in the presence of 2 mm IP_6_ allowed us to get better crystals grown in 0.2 m magnesium chloride, 0.1 m sodium acetate, pH 5.5, 16% (v/v) PEG 6000. Soaking experiments in precipitant solutions containing 10 mm IP_6_ or 10 mm IP_6_/ADP during 3 h yielded the complexes *m*IP_5_ 2-K/IP_6_ and *m*IP_5_ 2-K/IP_5_/ATP diffracting to 2.4 Å in the last case. Microseeding technique was necessary to improve the quality of all these crystals. For this purpose, we selected our best crystals and introduced them into 50 μl of crystallization solution plus a seeding bead. After two cycles of 30 s vortexing and 30 s on ice, we made a seed stock. We streak-seeded the crystallization drops with a whisker using this seed stock. All IP_5_ 2-K crystals appeared in a few hours after setting up the crystallization trials, and we observed that the protein is degraded in the crystallization conditions very quickly thus making crystal optimization extremely difficult.

### Data collection and structural determination

Crystals were transferred for a few seconds into precipitant solution plus 20% (v/v) glycerol and then flash-cooled in liquid nitrogen. Data from IP_5_ 2-K crystals were collected at 100 K in beam line BL13-XALOC of the ALBA Synchrotron (36). ΔC-mIP_5_ 2-K crystallizes in monoclinic P2_1_ space group in two different forms having one (pH 5.5) or two molecules (pH 6.25) in the asymmetric unit (Table 1). Diffraction data were indexed, integrated, and scaled using XDS (37) and merged using Aimless (38) from CCP4 suite (39, 40). Initially, ΔC-*m*IP_5_ 2-K monoclinic crystals grown at pH 6.5 (3.2 Å) allowed us to get a partial model using molecular replacement with MOLREP (41) and the structure of *A. thaliana* IP_5_ 2-K as a search model (Protein Data Bank code 2XAN). However, the preliminary electron density maps presented many ambiguities, although clearly showed high positive difference peaks for two possible zinc ions. An anomalous map computed with PHENIX (42) showed a strong anomalous signal in those positions (supplemental Fig. S2). Therefore, we tried SAD phasing in combination with MR (MRSAD-Auto-Rickshaw) (43). The heavy atom positions were located using PHASER (44) and refined with MLPHARE (39). The phases obtained were then combined, and density modification was performed with RESOLVE (45, 46) and PIRATE (47). Final electron density maps allowed the building of the whole chain except some exposed loops indicating the flexibility of these regions.

Later, the ΔC-*m*IP_5_ 2-K monoclinic crystals grown at pH 5.5 allowed us to refine the structure of protein complexes with ligands IP_6_ and IP_5_/ATP to 2.6 and 2.4 Å maximum resolution, respectively. The structures were solved by molecular replacement using MOLREP (41) and the coordinates of ΔC-*m*IP_5_ 2-K described above as a search model. The substrates/products were manually fit into the electron density maps. Although we soaked the crystals with the products IP_6_/ADP, the initial electron density maps showed clear density consistent with IP_5_ and ATP (supplemental Fig. S1). Then, we checked that our crystallized *m*IP_5_ 2-K samples are able to catalyze both the forward and reverse reactions in solution (Fig. 3, *B* and *C*), and, therefore, we modeled the substrates IP_5_/ATP in the active site. Model refinement was performed with REFMAC (48) alternating with manual model building using COOT (49). The stereochemistry of the model has been checked with PROCHECK (50). Statistics for all data processing and refinement are summarized in Table 1. Figures of the models were generated with PyMOL (51).

### Circular dichroism

CD spectra were recorded using a Jasco-810 spectropolarimeter equipped with a Peltier-thermostatted cell holder. Measurements in the far-UV region (250–200 nm) were performed using the samples WT and C291S ΔC-IP_5_ 2-K after protein buffer exchange to 25 mm sodium phosphate, pH 8, and at protein concentrations of 0.1 mg ml^−1^ (10-mm path length quartz cells; bandwidth, 1 nm; response, 4 s; scan speed, 20 nm min^−1^). Collected spectra were the average of four accumulations. The data were converted to molar ellipticities after subtraction of the buffer contribution using the average molecular mass per residue (114 Da). Thermal denaturation was monitored by CD measuring the ellipticity changes at 220 nm as the temperature was raised (20–90 °C) at 60 °C h^−1^. The normalized ellipticity value at each temperature was calculated as ([θ]*t* − [θ]25)/([θ]90 − [θ]25), where [θ]*t* is the ellipticity value at temperature *t*, and [θ]25 and [θ]90 are the ellipticity values at 25 and 90 °C, respectively.

### Protein sequence alignments and bioinformatics

IP_5_ 2-Ks sequences of all kingdoms have been retrieved using BLAST (https://blast.ncbi.nlm.nih.gov/Blast.cgi) and Pfam (52) searches. After removing all incomplete sequences or lacking essential hallmarks for kinase function, we had a collection of the following sequences: 102 for mammals, 142 for non-mammal vertebrates, 28 for invertebrates, 158 for fungi, and 203 for plants. Sequence alignments have been performed with the Clustal Omega server (53) and corrected manually with SeaView (54). A structural alignment between *At*IP_5_ 2-K (code 1xan) and *m*IP_5_ 2-K has been performed with EPSPRIT (55).

### Enzyme assays

LSL-ΔC-IP_5_ 2-K and ΔC-IP_5_ 2-K samples showed comparable activity (data not shown); therefore, we used LSL-tagged samples for analysis of kinetic parameters of WT and mutants. For this purpose, IP_5_-dependent conversion of ATP to ADP was determined by HPLC. Assays were performed in 20 mm Hepes, 1 mm MgCl_2_, pH 7.3, containing 0.2–5 μm Ins(1,3,4,5,6)P_5_ and 50 μm ATP in a volume of 50–100 μl at an enzyme concentration of 4 μg ml^−1^. Reactions were stopped by the addition of 50 μl of 60 mm (NH_4_)_2_HPO_4_, pH 3.8, with H_3_PO_4_. Aliquots of the reaction products were resolved by ion-pair reverse-phase chromatography (56) with the following modifications: separations were performed on a 100 × 2.1-mm Agilent X-Bridge C18 (3.5-μm particle size) column eluted at a flow rate of 0.25 ml min^−1^. Nucleotide substrates and products (ADP and ATP) were detected at 260 nm, and the extent of conversion of one to the other was determined from the ratio of integrated peaks. The ADP content of the ATP used was less than 0.1% of the ATP peak area. Reaction velocities were calculated assuming 1:1 stoichiometry of consumption of nucleotide and inositide. Experimental data were fitted by non-linear least squares regression to the Michaelis-Menten equation in GraFit (Erithacus Software). Assays were performed in triplicate and the experiment repeated an additional three times with similar results.

### Verification of reactions catalyzed by mIP_5_ 2-K

We undertook a variety of enzyme assays to determine the identities of products formed by the enzyme. We tested the ability of ΔC-*m*IP_5_ 2-K to catalyze forward “kinase” and reverse reactions.

For the forward kinase reaction, 2.4 μg of ΔC-*m*IP_5_ 2-K was incubated with 200 μl of 100 μm Ins(1,3,4,5,6)P_5_ (sodium salt, SiChem, Germany), 40 μm ATP in 20 mm Hepes, 1 mm MgCl_2_, pH 7.3, at 37 °C. At intervals, aliquots were withdrawn and 20 μl injected onto a 250 × 3-mm CarboPac PA200 column (Dionex) eluted at a flow rate of 0.4 ml min^−1^ with a gradient derived from buffer reservoirs containing the following: A, water; B 0.6 m methanesulfonic acid according to the following profile: time (min), % B; 0, 0; 25, 100; 38, 100. The eluate from the column was mixed in a mixing tee with color reagent (0.1% w/v Fe(NO_3_)_3_·9H_2_O in 2% v/v HClO_4_) (57) delivered at a flow rate of 0.2 ml min^−1^ by a second HPLC pump. The combined flow was monitored at 290 nm after passage through a knitted reaction coil.

For the reverse reaction, 2.4 μg of ΔC-*m*IP_5_ 2-K was incubated with 200 μl of 100 μm IP_6_ (sodium salt, Merck, Germany), 50 μm ADP in 20 mm Hepes, 1 mm MgCl_2_, pH 7.3, at 37 °C, with subsequent processing as above. The identity of IP_5_ and IP_6_ products was confirmed by chromatography of standards (SiChem or Merck) and by analysis of an IP_6_ hydrolysate obtained by overnight refluxing of IP_6_ in 1 m HCl, with subsequent rotary evaporation to remove HCl.

Our Ins(1,3,4,5,6)P_5_ (IP_5_ 2-OH) substrate contained d- and/or l-Ins(1,2,4,5,6)P_5_ (IP_5_ 1/3-OH), but our analysis which resolves the two *meso*-compounds (IP_5_ 2-OH and IP_5_ 5-OH) from the two pairs of enantiomers (IP_5_ 1/3-OH and IP_5_ 4/6-OH) confirmed that *m*IP_5_ 2-K is an inositol 1,3,4,5,6-pentakisphosphate 2-kinase that does not accept d- and/or l-Ins (1,2,4,5,6)P_5_ (mixture unknown) as substrate (Fig. 3*B*). HPLC traces were exported from Jasco (Great Dunmow, UK) ChromNav software as asci files and redrawn in GraFit (Erithacus Software).

### Inductively coupled plasma-optical emission spectroscopy

Metal analysis of *m*IP_5_ 2-K was performed by optical emission spectroscopy on a Varian Vista Pro ICP-OES. Protein or buffer in which protein was prepared was diluted 125–139-fold in 18.2 megohm·cm water containing 1 n HNO_3_. Diluted protein or buffer was subjected to ICP-OES on a machine calibrated with 0–4.0 μm standards of cobalt, copper, nickel, and zinc in 1 n HNO_3_. All metals except zinc were close to the limit of detection in the diluted protein sample; they were only slightly above the background in the buffer (supplemental Table S2).

## Author contributions

E. F.-E and J. M. G. prepared the constructs for the experiments. E. F.-E expressed, purified, and crystallized all the protein samples. E. F.-E and B. G. solved the protein structures. E. F.-E., J. S.-A., and B. G. analyzed the structural data. C. A. B. performed all enzyme assays and zinc measurements. J. S.-A. participated in the work and results and discussion. B. G. designed the research and wrote the paper. All authors edited the manuscript.

## Supplementary Material

Supplemental Data

## Footnotes

3 The abbreviations used are:

IP_5_ 2-K: inositol 1,3,4,5,6-pentakisphosphate 2-kinase
IP: inositol phosphate
IP_5_: inositol 1,3,4,5,6-pentakisphosphate
IP_6_: inositol hexakisphosphate
IPK: inositol polyphosphate kinase
LSL: *Laetiporus sulphurous* lectin
PEG: polyethylene glycol
TEV: tobacco etch virus
UBF: upstream binding factor
IP_3_: inositol 1,4,5-trisphosphate
ICP-OES: inductively coupled plasma-optical emission spectroscopy
*m*IP: mouse IP
*h*IP: human IP
r.m.s.d.: root mean square deviation
CL: connecting loop
Ins(1,2,4,5,6)P_5_: 1,2,4,5,6-inositol pentakisphosphate
Ins(1,3,4,5,6)P_5_: inositol 1,3,4,5,6-pentakisphosphate.
