# Supplementary material for: The crystal structure of mammalian inositol 1,3,4,5,6-pentakisphosphate 2-kinase reveals a new zinc-binding site and key features for protein function
Source: J Biol Chem. 2017 Apr 27;292(25):10534–48. doi: 10.1074/jbc.M117.780395 (PMC5481561; doi:10.1074/jbc.M117.780395)
Supplement: Supplemental Data [file supp_292_25_10534__index.html]

The Crystal Structure of Mammalian Inositol 1,3,4,5,6-Pentakisphosphate 2-Kinase Reveals a New Zinc Binding Site and Key Features for Protein Function — The crystal structure of mammalian inositol 1,3,4,5,6-pentakisphosphate 2-kinase reveals a new zinc-binding site and key features for protein function — Structure of a mammal IP5 2-K — Supplemental Data 

# The crystal structure of mammalian inositol 1,3,4,5,6-pentakisphosphate 2-kinase reveals a new zinc-binding site and key features for protein function

## Supplemental Data

- Supplemental Data (.pdf, 6.1 MB)
