# Supplementary material for: The crystal structure of mammalian inositol 1,3,4,5,6-pentakisphosphate 2-kinase reveals a new zinc-binding site and key features for protein function
Source: J Biol Chem. 2017 Apr 27;292(25):10534–48. doi: 10.1074/jbc.M117.780395 (PMC5481561; doi:10.1074/jbc.M117.780395)
Supplement: Supplemental Data [file 10.1074_M117.780395_jbc.M117.780395-1.pdf]

SUPPLEMENTAL DATA

The Crystal Structure of Mammalian Inositol 1,3,4,5,6-Pentakisphosphate 2-Kinase Reveals a New Zinc Binding Site and Key Features for Protein Function

Elsa Franco-Echevarría[a], Julia Sanz-Aparicio[a], Charles A. Brearley[b], Juana M[a] González-Rubio[a] and Beatriz González [a#]

a *Departamento de Cristalografía y Biología Estructural, Instituto de Química-Física "Rocasolano," CSIC, Serrano 119, 28006-Madrid, Spain*

b *School of Biological Sciences, University of East Anglia, Norwich Research Park, Norwich NR4 7TJ, United Kingdom*

TABLE OF CONTENTS

**Figure S1**	Electron density map of *m*IP_5 2-K ternary and binary complexes.
**Figure S2**	Anomalous electron density map of *m*IP_5 2-K
**Table S1**	*m*IP_5 2-K residues involved in ligands binding through polar contacts < 4 Å.
**Table S2**	Metal content of LSL-mIP5 2-K measured by ICPOES
**Table S3**	DNA primers used for the ΔC-*m*IP_5 2-K constructs prepared in this work

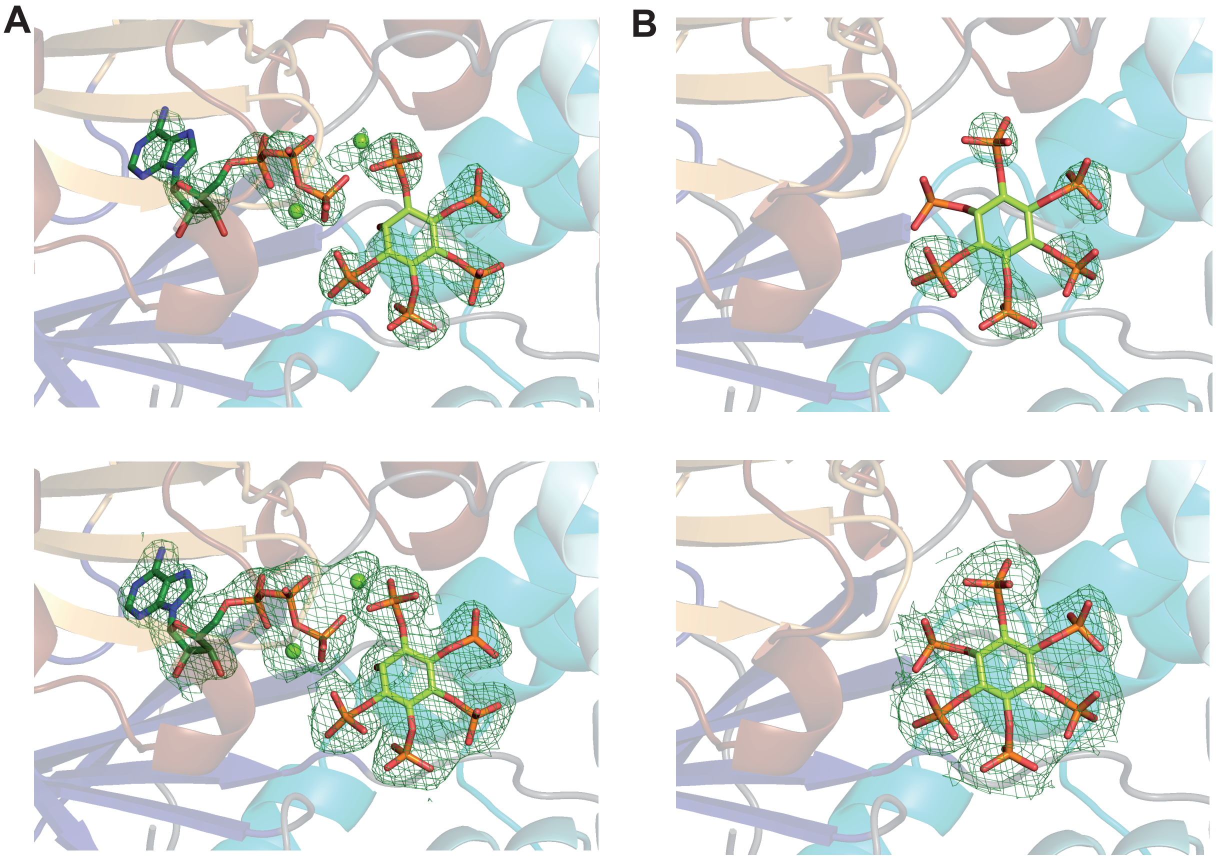

Figure S1. Electron density map of *m*IP$_5$ 2-K ternary and binary complexes.
A, electron density map for the ternary complex. Protein and ligands (ATP and IP$_5$) are shown as cartoon and sticks representation respectively. Mg ions are shown as green spheres. The upper panel shows the composite omit map Fo-Fc using the annealing protocol and the torsion method, and contoured at 3σ level. The lower panel corresponds to the σ-weighted 2Fo–Fc electron density map contoured at 1.0 σ. *B*, Same as A for the binary complex, showing IP$_6$.

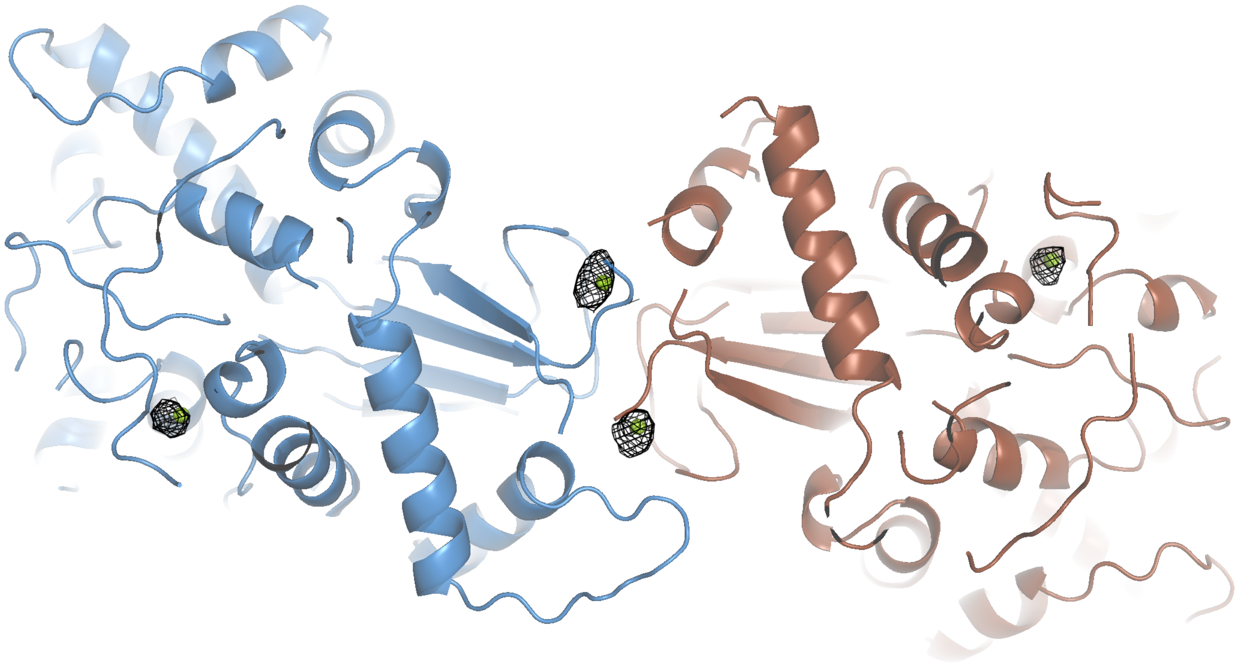

Figure S2. Anomalous electron density map of *m*IP_5 2-K
Anomalous electron density map for the *m*IP_5 2-K crystals obtained at 3.2 Å. The two molecules of the asymmetric unit are represented in different colours. The four Zinc atoms are shown as green spheres. The electron density shown corresponds to the anomalous 2Fo-Fc map contoured at 4σ level.

Table S1. *m*IP_5 2-K residues involved in ligands binding through polar contacts < 4 Å.

IP_5	d (Å)	IP_5 ligand
P1	4.0	N18 (N)
P1	2.7	R100 (NH1)
P1	3.3	R100 (NH2)
P1	3.3	**K138 (NZ)**
P1	3.4	K173 (NZ)
P1	2.8	N206 (ND2)
OH2	2.8	**K138(NZ)**
OH2	2.7	**D400 (OD1)**
OH2	2.7	**D400 (OD2)**
P3	3.4	N18 (ND2)
P3	4.0	K19N
P3	3.6	K19 (NZ)
P3	3.1	K441 (NZ)
P3	2.8	Mg1
P4	2.6	Q449 (NE2)
P5	3.4	K140(NZ)
P5	2.6-W_1-3.2	R160 (NE)
P5	3.6	R160 (NH2)
P5	3.1-W_1-2.7	H164 (ND1)
P6	2.9	**K138 (NZ)**
P6	3.3	K140 (NZ)
P6	2.8,3.4	K168 (NZ)
P6	3.1,3.3	N206 (ND2)
P6	3.0-W_2-2.8	N206 (OD1)
P6	2.9-W_2-2.8	N207 (OD1)

ATP	d (Å)	ATP ligand
N1	3.0	L118 (N)
N3	3.4	H14 (ND1)
N6	2.8	P116 (O)
O2´	2.5	E136 (OE2)
O3´	2.7	E136 (OE1)
O3´	3.8	R209 (NH1)
Pα, Pβ	2.8,3.2	S20 (OG)
Pα, Pβ	2.9	R33 (NH2)
Pβ	3.4	G17 (N)
Pβ	3.1	K19 (N)
Pβ	2.9	S20 (N)
Pγ	3.3	N18 (N)
Pγ	3.5	K138 (NZ)
Pγ	3.3-W_3-3.0	N206 (ND2)
Pγ	3.2	**D400 (OD2)**
Pβ, Pγ	2.6,2.6	Mg1
Pα, Pβ, Pγ	2.0, 2.8, 2.2	Mg2

Mg	d (Å)	Mg ligand
Mg1	2.4	**D437 (OD1)**
Mg1	3.7,3.7	**D439 (OD1, OD2)**
Mg1	2.6,2.6	Pβ, Pγ ATP
Mg1	2.8	P3 IP5
Mg2	2.3	**D437 (OD2)**
Mg2	2.4-W_4-3.0,2.6	**D400 (O**), S402 (OG**)**
Mg2	2.0,2.8,2.2	Pα, Pβ, Pγ ATP

Notes:
W: water
Bold letter: catalytic residues
P: phosphate group

Table S2. Metal content of LSL-mIP$_5$ 2-K samples measured by ICPOES

*m*IP$_5$ 2-K samples	[Zn] /SD* 213.857 nm	[Co] /SD 228.615 nm	[Ni]/SD 230.299 nm	[Cu]/SD 327.395 nm	[Cu]/SD 324.754 nm	[Ni]/SD 231.604 nm	[Fe]/SD 259.940 nm	[Mn]/SD 259.372 nm
	μM	μM	μM	μM	μM	μM	μM	μM
WT	17,761/ 0,226	0,445/ 0,062	0,520/ 0,168	0,468/ 0,024	0,301/ 0,011	0,679/ 0,099	0,899/ 0,179	0,028/ 0,002
H129S	13,978/ 0,306	0,172/ 0,050	1,646/ 0,338	0,564/ 0,098	0,336/ 0,015	-0,050/ -0,033	0,245/ 0,080	-0,026/ 0,004
C291S	4,086/ 0,113	0,772/ 0,150	0,622/ 0,355	0,308/ 0,044	0,034/ 0,001	-0,019/ -0,006	0,666/ 0,240	3,897/ 0,128
C410S	11,478/ 0,266	0,623/ 0,092	1,173/ 0,358	0,393/ 0,031	0,608/ 0,015	-0,746/ -0,515	0,497/ 0,169	0,128/ 0,008

*** Mean of metal concentration (experiment performed in triplicate)/standard deviation**

*LSL-m*IP$_5$ 2-K *sample*		Zinc	Protein: Zinc molar ratio
	Concentration μM	Concentration μM	
WT	30,5	17,8	1:0.6
H129S	16,7	14.0	1:0.8
C291S	11,1	4,1	1:0.4
C410S	16,7	11,5	1:0.7

NOTE: values with buffer discount of 0.28 microM

Table S3. DNA primers used for the ΔC-*m*IP$_5$ 2-K constructs prepared in this work.

Construct	N.	F/R[1]	Primer sequence 5´-3	Cloning sites	PCR template
ΔCmIP52K/pKLSLt	1	F	CATGCCAAAGAT**TAG**ACTGTGAGGTCG	EcorI/HindIII. Stop codón at 470	mIP52K/PKLSL
	2	R	CGACCTCACAGT**CTA**ATCTTTGGCATG		
N18G/K19A-ΔCmIP52K/pKLSLt	3	F	CATGGCGAAGGC**GGCGCG**AGCCTTGTGGTG	EcorI/HindIII	ΔCmIP52K/pKLSLt
	4	R	CACCACAAGGCT**CGCGCC**GCCTTCGCCATG		
R100A-ΔCmIP52K/pKLSLt	5	F	GACCAGAGTCC**GCT**TGTGACAAGGAC	EcorI/HindIII	ΔCmIP52K/pKLSLt
	6	R	GTCCTTGTCACA**AGC**GGACTCTGGTC		
H129S-ΔCmIP52K/pKLSLt	7	F	CACTTTGCAGAG**AGC**CGGCCGATTCTG	EcorI/HindIII	ΔCmIP52K/pKLSLt
	8	R	CAGAATCGGCCG**GCT**CTCTGCAAAGTG		
K138A-ΔCmIP52K/pKLSLt	9	F	CCCACATTTTGG**CGC**AATCTCTACACAC	EcorI/HindIII	ΔCmIP52K/pKLSLt
	10	R	GTGTGTAGAGATT**GCG**CCAAAATGTGGG		
K173A-ΔCmIP52K/pKLSLt	11	F	GGTAGCAACTGGA**GCG**TGGAAGAAAATC	EcorI/HindIII	ΔCmIP52K/pKLSLt
	12	R	GATTTTCTTCCA**CGC**TCCAGTTGCTACC		
C181S-ΔCmIP52K/pKLSLt	13	F	TAAATAC**AGC**CCCCTCGACCTCTACTCAGGAAATAA	EcorI/HindIII	ΔCmIP52K/pKLSLt
	14	R	TTATTTCCTGAGTAGAGGTCGAGGGG**GCT**GTATTTA		
L281A-ΔCmIP52K/pKLSLt	15	F	CCCGAGCTGGTGCC**GCA**AGGCTTGGGCTCCAGGGCCCA	EcorI/HindIII	ΔCmIP52K/pKLSLt
	16	R	TGGGCCCTGGAGCCCAAGCCT**TGC**GGCACCAGCTCGGG		
L281A/L283A-ΔCmIP52K/pKLSLt	17	F	CCCGAGCTGGTGCC**GCA**AGG**GCT**GGGCTCCAGGGCCCA	EcorI/HindIII	L281A-ΔCmIP52K/pKLSLt
	18	R	TGGGCCCTGGAGCCC**AGC**CCT**TGC**GGCACCAGCTCGGG		
C291S-ΔCmIP52K/pKLSLt S	19	F	GGGCCCACGAGTC**AGC**GAAGCCAGTCC	EcorI/HindIII	ΔCmIP52K/pKLSLt
	20	R	GGACTGGCTTC**GCT**GACTCGTGGGCCC		
Y363A-ΔCmIP52K/pKLSLt	21	F	GATGGGCCT**GCT**GATGAAGTATTTTACC	EcorI/HindIII	ΔCmIP52K/pKLSLt
	22	R	GGTAAAATACTTCATC**AGC**AGGCCCATC		
C410S-ΔCmIP52K/pKLSLt	23	F	GCACTTTCCCCT**AGC**CTACAGGGCACC	EcorI/HindIII	ΔCmIP52K/pKLSLt
	24	R	GGTGCCCTGTAG**GCT**AGGGGAAAGTGC		
D437A-ΔCmIP52K/pKLSLt	25	F	GTCTGTATTG**GCC**CTCGACCTCAAGC	EcorI/HindIII	ΔCmIP52K/pKLSLt
	26	R	GCTTGAGGTCGAG**GGC**CAATACAGAC		
D439A-ΔCmIP52K/pKLSLt	27	F	GTATTGGACCTC**GCC**CTCAAGCCCTATG	EcorI/HindIII	ΔCmIP52K/pKLSLt
	28	R	CATAGGGCTTGAG**GGC**GAGGTCCAATAC		

* bold letter marks the position of the mutated codon.
[1]F/R=Forward/Reverse
